# Evaluation of Recombinant Herpesvirus of Turkey Laryngotracheitis (rHVT-LT) Vaccine against Genotype VI Canadian Wild-Type Infectious Laryngotracheitis Virus (ILTV) Infection

**DOI:** 10.3390/vaccines9121425

**Published:** 2021-12-03

**Authors:** Catalina Barboza-Solis, Shahnas M. Najimudeen, Ana Perez-Contreras, Ahmed Ali, Tomy Joseph, Robin King, Madhu Ravi, Delores Peters, Kevin Fonseca, Carl A. Gagnon, Frank van der Meer, Mohamed Faizal Abdul-Careem

**Affiliations:** 1 Health Research Innovation Center 2C53, Faculty of Veterinary Medicine, University of Calgary, 3330 Hospital Drive NW, Calgary, AB T2N 4N1, Canada; catalina.barboza@ucalgary.ca (C.B.-S.); fathimashahnas.moham@ucalgary.ca (S.M.N.); ana.perezcontreras@ucalgary.ca (A.P.-C.); ahmed.ali@ucalgary.ca (A.A.); fjvander@ucalgary.ca (F.v.d.M.); 2 Department of Pathology, Beni-Suef University, Beni Suef 62511, Egypt; 3 Animal Health Centre, Ministry of Agriculture, Food and Fisheries, Abbotsford, BC V3G 2M3, Canada; tomy.joseph@gov.bc.ca; 4 Agri Food Laboratories, Alberta Agriculture and Forestry, Edmonton, AB T6H 4P2, Canada; blking@telus.net; 5 Animal Health and Assurance, Alberta Agriculture and Forestry, Edmonton, AB T6H 4P2, Canada; madhu.ravi@gov.ab.ca (M.R.); delores.peters@gov.ab.ca (D.P.); 6 Provincial Laboratory for Public Health, Calgary, AB T2N 4W4, Canada; kevin.fonseca@albertaprecisionlabs.ca; 7 Swine and Poultry Infectious Diseases Research Center (CRIPA), Faculty of Veterinary Medicine, University of Montreal, 3200 Sicotte, Saint-Hyacinthe, QC J2S 2M2, Canada; carl.a.gagnon@umontreal.ca

**Keywords:** infectious laryngotracheitis virus, rHVT-LT recombinant vaccine, chicken, immune response

## Abstract

In Alberta, infectious laryngotracheitis virus (ILTV) infection is endemic in backyard poultry flocks; however, outbreaks are only sporadically observed in commercial flocks. In addition to ILTV vaccine revertant strains, wild-type strains are among the most common causes of infectious laryngotracheitis (ILT). Given the surge in live attenuated vaccine-related outbreaks, the goal of this study was to assess the efficacy of a recombinant herpesvirus of turkey (rHVT-LT) vaccine against a genotype VI Canadian wild-type ILTV infection. One-day-old specific pathogen-free (SPF) White Leghorn chickens were vaccinated with the rHVT-LT vaccine or mock vaccinated. At three weeks of age, half of the vaccinated and the mock-vaccinated animals were challenged. Throughout the experiment, weights were recorded, and feather tips, cloacal and oropharyngeal swabs were collected for ILTV genome quantification. Blood was collected to isolate peripheral blood mononuclear cells (PBMC) and quantify CD4+ and CD8+ T cells. At 14 dpi, the chickens were euthanized, and respiratory tissues were collected to quantify genome loads and histological examination. Results showed that the vaccine failed to decrease the clinical signs at 6 days post-infection. However, it was able to significantly reduce ILTV shedding through the oropharyngeal route. Overall, rHVT-LT produced a partial protection against genotype VI ILTV infection.

## 1. Introduction

*Gallid herpesvirus-1* (GaHV-1) or infectious laryngotracheitis virus (ILTV, by its historic nomenclature) is an alphaherpesvirus belonging to the genus *Iltovirus* and family *Herpesviridae*, which causes infectious laryngotracheitis (ILT) in chickens [1]. There is also published evidence that shows that ILTV is capable of infecting pheasants, peafowls and turkeys [2,3,4,5]. ILT is an acute upper respiratory disease that impacts mainly chickens reared in high-density populations [6]. The transmission of ILTV is horizontal [7]. The pathogenicity of the virus is dependent on the isolate, age of the bird and the infective dose [6]. Chickens of all ages are susceptible to ILTV infection, with reports as early as 3 weeks of age [8]. Some of the clinical signs observed are conjunctivitis, nasal discharge, drop in egg production and reduced weight gain [6]. As a herpesvirus, ILTV can establish latency mainly in the trigeminal ganglion [9] and to a lesser degree in trachea [10].

In 1925, the first case of ILT was reported in Canada [2]; since then, outbreaks in backyard and commercial poultry flocks in the country have been reported [11,12,13,14,15,16,17]. In Ontario (ON) and Alberta (AB), ILT is endemic in backyard poultry flocks that are known to house multi-age chickens and generally have low biosecurity standards. These practices allow ILTV establishment and maintenance, causing a continuous risk for commercial poultry operations [18]. To tackle ILT, a combination of biosecurity measures and vaccination strategies were implemented.

The live attenuated vaccine was the first of its type to be commercially available against ILT [19]. Based on the method of attenuation, there are two types of live attenuated vaccines: the chicken embryo origin (CEO), derived from consecutive passages in embryonated chicken eggs [19], and the tissue culture origin (TCO), produced by consecutive passages of the ILTV ASL L-6 strain in primary avian-origin cells [20]. Some limitations of the live attenuated ILT vaccines are their ability to regain virulence following bird-to-bird passage [21], and establish latency [22]. CEO vaccine-related ILTV strains cause frequent ILT outbreaks in North America [11,13,23]. Additionally, there has been a surge in ILTV recombination involving CEO or TCO vaccinal ILTV strains [11,12,24,25,26]. This has resulted in ILT vaccine failures, leading to ILT outbreaks in vaccinated flocks [27].

There have been several outbreaks of ILT in recent years, predominantly involving CEO revertant and wild-type strains in Canada [11,12,28]. In AB, outbreaks are more commonly seen in backyard flocks where only 13% are vaccinated against ILTV [29]. In addition to the live attenuated vaccines, in AB, recombinant vaccines are recommended to be administered at hatchery in ovo or post-hatch. These viral vector vaccines replicate to a similar degree in the secondary lymphoid organs of chickens, such as the spleen, regardless of the application route [30]. There are three recombinant viral vector vaccines licensed in Canada. One uses fowlpox virus (rFPV) as a vector, and the other two use a turkey herpesvirus (rHVT) as a backbone. The rFPV-LT vaccine uses glycoprotein (g) B and UL-32 genes of ILTV as inserts (Vectormune^®^ FP LT+AE, CEVA Biomune, Lenexa, KS, USA) [31]. On the other hand, both rHVT-LTs use ILTV gI and gD (Innovax^®^ ILT, Merck Animal Health, Summit, NJ, USA) [30] or the gB and UL-32 genes (Vectormune^®^ HVT LT, CEVA Biomune, Lenexa, KS, USA) [32] as inserts. Some of the advantages of these vaccines are the prevention of bird-to-bird transmission [32] and lack of reversion to virulence [33].

The efficacy of rHVT-LT and rFPV-LT vaccines is variable [32,34,35,36,37,38]. It has been suggested that rHVT-LT has a slower onset of immunity [32,35] and is most efficacious after 7 weeks of age [32]. This prompted us to investigate if an immune response is induced when the vaccinated chickens are infected at 3 weeks post-vaccination, since ILTV can occur at this early age. Previous studies have only investigated the humoral immune response, where results showed no correlation between antibodies and protection elicited by vaccination [34,35,36]. It has been noted that performing vaccine evaluation studies using field-derived challenge strains closely mimics field conditions [27]. At this stage, there are no experimental data on the protective efficacy of the rHVT-LT vaccine against Canadian ILTV field strains. In this study, we evaluated if 1-day-old chickens vaccinated with a rHVT-LT vaccine were sufficiently protected against a challenge with a wild-type Canadian ILTV isolate at 3 weeks post-vaccination.

## 2. Materials and Methods

### 2.1. Animals and Ethics Statement

The Health Science Animal Care Committee (HSACC) of the University of Calgary, AB, Canada (Protocol number: AC19-0013), approved the use of specific pathogen-free (SPF) eggs, embryos and live chickens. The SPF eggs and chickens were obtained from the Canadian Food Inspection Agency, Ottawa, Canada. The ILTV-infected chickens were maintained in high containment poultry isolators (Plas Labs^TM^, Fisher scientific, Ottawa, ON, Canada) at the Prion/Virology Facility at the Foothills Campus of the University of Calgary. Mock-infected chickens were kept in the Animal Resource Center (ARC) at the Foothills Campus of the University of Calgary. The experimental animals were provided with ad libitum access to food and water, and veterinary care was provided by resident veterinarians.

### 2.2. ILTV Virus

ILTV AB-S63, which belongs to genotype VI [11], was obtained from the Agri Food Laboratories (Alberta Agriculture and Forestry, Edmonton, AB, Canada). This ILTV strain was isolated from an ILT outbreak in backyard chicken flock in AB, Canada [12]. The virus was propagated in the chorioallantoic membrane (CAM) of 10-day-old SPF chicken embryos as previously described [11]. The virus stock was titrated in chicken embryo liver cells (CELIC) prepared from 14-day-old chicken embryos as previously described [39]. The median tissue culture infective dose (TCID_50_) was calculated following the Reed and Muench method [40]. Previously, it has been shown that ILTV AB-S63 was virulent and induced ILT in SPF chickens [39].

### 2.3. Vaccine and Vaccine Titration

The recombinant vaccine, rHVT-LT (Innovax^®^ ILT, Merck Animal Health, Summit, NJ, USA), was used to vaccinate the chickens. The vaccine was titrated in a secondary chicken embryo fibroblast (CEF) monolayer in 60 mm plates. Ten-fold dilutions of the reconstituted vaccine were produced in Dulbecco’s modified Eagle medium (DMEM), and 200 μL of each dilution was added to three plate replicates. The inoculated cells were incubated at 37 °C and 5% CO_2_ for 5 days. At day 5 following inoculation, plaques were microscopically counted. Virus titer was calculated and recorded as 6360 plaque forming units (PFU)/per dose.

### 2.4. Experimental Design

The SPF eggs were incubated in digital egg incubators (Kingsuromax 20 and Rcom MARU Deluxe max, Autoelex Co., Ltd., GimHae, GyeongNam, Korea) according to the manufacturer’s instructions. Upon hatching, 44 1-day-old chickens were randomly assigned to four different groups (*n* = 11 per group). Two groups of chickens were vaccinated with a full dose of rHVT-LT subcutaneously, as prescribed by the manufacturer. The other two groups were mock vaccinated with the vaccine diluent provided by the manufacturer (Merck Animal Health, Summit, NJ, USA). At three weeks of age, one group of the vaccinated and one group of the mock-vaccinated chickens were infected with the ILTV AB-S63 strain. A dose of 10^3.5^ TCID_50_ in a total volume of 200 μL per chicken (100 μL was delivered via intratracheal administration and 50 μL per eye mucosal surface) [39]. The other two groups were mock infected with phosphate buffered saline (PBS) (Table 1).

Following ILTV infection, the chickens were observed twice a day for 14 days to record clinical signs. The clinical signs were scored with a value from 1 to 4 depending on their severity, as described previously [39]. Briefly, the clinical signs of ruffled feathers, droopy wings, depression, and bodyweight loss received a score of 1. The clinical signs of increased respiratory rate with an open beak and conjunctivitis received a score of 2. A severe increase in respiratory rate, marked by gasping and bloody mucus expectoration, received a score of 3 or 4, respectively. The obtained clinical sign scores were added together to obtain a cumulative clinical score per day. A cumulative clinical score of 4 was considered the humane end point. Oropharyngeal and cloacal swabs were collected at 3, 7, 10 and 14 days post-infection (dpi). All the collected swabs were placed in 1 mL of viral transport medium (Dulbecco’s modified Eagle medium (DMEM) supplemented with 3% fetal bovine serum (FBS), 0.02M HEPES and 0.25 mg/mL of penicillin and streptomycin (Gibco, Carlsbad, CA, USA). At the same time points, body weights were recorded, and feather tips were collected for DNA extraction (RNA Save, BI, Cromwell, CT, USA). The samples were stored at −80 °C until processed. At 5 and 12 dpi, 1.5 mL of blood was collected from the jugular vein with syringes previously filled with sodium citrate [41]. The samples were stored on ice until processed for PBMC isolation. At 14 dpi, all the chickens were euthanized, and post-mortem examination was performed to record gross lesions in the respiratory tissues. Trachea and lung samples were collected in 10% buffered formalin (VWR International, Radnor, PA, USA) for histological examination. Additionally, the trachea, lung and spleen were collected in RNA Save (Biological Industries, FroggaBio, Toronto, ON, Canada) for ILTV genome load quantification and HVT genome load quantification. At the end of the experiment, serum samples were also collected for the quantification of anti-ILTV antibodies.

### 2.5. Serology

The blood samples were kept overnight at room temperature, and serum was collected following centrifugation at 2500 rounds per minute (rpm) for 15 min (4 °C). The serum samples were analyzed for the quantification of anti-ILTV antibodies using a commercial enzyme-linked immunosorbent assay (ELISA) kit (ProFLOCK LT ELISA Kit; Synbiotics Corp., San Diego, CA, USA). The sample to positive value (*s/p*) and titer calculations were performed according to the manufacturer’s instructions. This kit does not detect antibodies produced by the rHVT-LT vaccine [30].

### 2.6. DNA Extraction and Quantitative Polymerase Chain Reaction (qPCR) Assay

DNA extraction from swabs, feather tips and tissues (trachea, lung and spleen) were performed using the QIAamp^®^ DNA Mini Kit (Qiagen, Hilden, Germany) based on the manufacturer’s instructions. The extracted DNA was quantified with the Nanodrop 1000 spectrophotometer (Thermo Scientific, Wilmington, DE, USA). The qPCR assay was carried out using a CFX96-c1000 Thermocycler (Bio Rad laboratories, Mississauga, ON, Canada), as previously described [42]. For the ILTV genome load quantification, the primers targeted the proteinase K (PK) gene. For the genome load quantification of the vaccine, the primers targeted the glycoprotein B (gB) gene of HVT (F: 5′-GCC AGT TGG ATA TCT GCC G-3′ and R: 5′-CGG CCA ATC ATC GTA GGT AC-3′). Additionally, the β-actin gene (F: 5′–CAA CAC AGT GCT GTC TGG TGG TA–3′ and R: 5′–ATC GTA CTC CTG CTT GCT GAT CC–3′) was used in each sample to normalize variation in the template amount. The total volume per reaction was 20 μL; this included DNA as a template (20 ng for swabs, 200 ng for tissues and 100 ng for feather tips), 10 pmol/μL of forward and reverse primer, 10 μL of SYBR Green Master Mix (Invitrogen, Burlington, ON, Canada) and DNAse/RNAse-free water (Thermo Scientific, Wilmington, DE, USA). Thermocycler conditions for PK and β-actin genes were as follows: 95 °C for 20 s (s) for initial denaturation, then 40 cycles of denaturation at 95 °C for 3 s, annealing at 57 °C for 30 s and elongation at 95 °C for 10 s. Thermocycler conditions for the gB gene were as follows: 95 °C for 20 s for initial denaturation, then 40 cycles of denaturation at 95 °C for 3 s, annealing at 60 °C for 30 s and elongation at 95 °C for 10 s.

### 2.7. Peripheral Blood Mononuclear Cell (PBMC) Isolation

The collected whole blood (1.5 mL) samples were diluted in 2.5 mL of Hank’s balanced salt solution (HBSS) and carefully layered into 15 mL SepMate^TM^ tubes (StemCell Technologies, Vancouver, BC, Canada) containing 3.5 mL Ficoll Plaque Premium (GE Healthcare, Chicago, IL, USA). The tubes were centrifuged for 15 min (min) at 1200× *g*, 20 °C. The upper layer consisting of PBMCs was decanted into a 15 mL falcon tube containing 7 mL of HBSS. Following mixing, the tubes were centrifuged at 400× *g* (18 °C) for 15 min. Following centrifugation, the supernatant was discarded, and the pellet was resuspended in 7 mL of HBSS. The resuspended cells were centrifuged again under the same conditions. Then, the supernatant was discarded, and the pellet was resuspended in Roswell Park Memorial Institute medium (RPMI; Gibco, Carlsbad, CA, USA) containing 1% l-glutamine, 1% of antibiotic (100 U/mL penicillin and 100 µg/mL streptomycin) and 10% fetal bovine serum (FBS, Gibco, Carlsbad, CA, USA). The PBMCs in each sample were counted using LUNA^TM^ Cell Counting Slides (Logos Biosystems, Annandale, VA, USA) with the Luna^TM^ Automated Cell Counter (Logos Biosystems, Annandale, VA, USA).

### 2.8. Staining for Flow Cytometry Analysis

The obtained PBMCs were strained with monoclonal antibodies as previously described [43]. Briefly, cells were washed with 1% bovine serum albumin (BSA) fraction V (OmniPur, EMD, Darmstadt, Germany) made in PBS and centrifuged at 211× *g* for 10 min at 4 °C. They were then suspended in 100 μL of 1:100 chicken serum (diluted in 1% BSA) for Fc blocking. After 10 min, the plate was centrifuged at 211× *g* for 10 min at 4 °C, and the cells were resuspended and incubated in the dark with phycoerythrin (PE)-conjugated mouse anti-chicken CD4 (Southern Biotech, Birmingham, AL, USA) and fluorescein isothiocyanate (FITC)-conjugated mouse anti-chicken CD8 (Southern Biotech, Birmingham, AL, USA). Their respective isotypes were used as controls, and 1% BSA was used for the unstained control. Samples were incubated in the dark for 30 min and spun at 211× *g* for 10 min (4 °C). The supernatant was discarded, and the pellet was resuspended in 1% paraformaldehyde [44] and submitted to the University of Calgary’s Flow Cytometry Core Facility (Calgary, AB, Canada). Flow cytometry was performed with a D LSR 11 (BD Bioscience, San Jose, CA, USA) with 4 lasers: violet, blue, yellow-green and red laser. Data analysis was performed with BD FACSDiva software version 6.1.3 (BD Bioscience, San Jose, CA, USA).

### 2.9. Histology

Tissue samples of the trachea preserved in 10% formalin were submitted to the Diagnostic Services Unit (DSU) of the University of Calgary Faculty of Veterinary Medicine (UCVM) to produce hematoxylin and eosin (H&E) stained sections.

### 2.10. Data and Statistical Analyses

The ILTV and HVT viral load quantification was based on standard curves of the PK and gB gene plasmids; in both cases, the β-actin gene was used as a housekeeping gene. For absolute quantification of the genome loads, threshold cycle (Ct) values were plotted against the standard curve data. To determine the starting quantities, the following formula was used:

(1) $$\mathrm{Log}\;\mathrm{starting}\;\mathrm{quantity}=\frac{\left[Ct-intercept\right]}{m}$$

(2) $$\mathrm{Starting}\;\mathrm{quantity}=10^{\mathrm{logstarting}\;\mathrm{quantity}}$$

*m* = Y − C of the standard curve.

The ELISA data processing, sample to positive value (*s*/*p* value) and titer calculations were performed according to the manufacturer’s instructions. Serum samples with a *s*/*p* ratio value of ≤0.150 received a “0” titer value and were considered negative for ILT antibody.

The tracheal histopathology was scored by a scale (0–5) developed by Guy and colleagues [45]. In this scale, a score of 0 represents normal tissue (thin pseudostratified columnar epithelium and normal mucous glands), a score of 1 represents minimal changes (normal epithelium with mild to moderate infiltration of lymphocytes, rare heterophils, normal mucous glands, no syncytia or cells with intranuclear inclusion bodies), a score of 2 represents mild changes (mucosa thickened due to mild to moderate cell infiltration and/or an essentially normal epithelium, except for foci of syncytia with intranuclear inclusion bodies; hyperemia, occasionally with cell cuffs), a score of 3 represents moderate changes (mucosa thickened due to moderate to marked cell infiltration; numerous syncytia with intranuclear inclusion bodies; patches of affected epithelium often separating from or, less commonly, sloughed from the lamina propria; mucosal surface well covered by normal or affected epithelium; mucous glands reduced; marked hyperemia; cuffs of mononuclear cells around vessels outside the mucosa), a score of 4 represents severe changes (mucosa thickened due to edema, proteinaceous fluid, cellular exudate or adherent fibrinohemorrhagic to cellular pseudo membrane on the surface; normal epithelium absent; mucosal surface covered by a thin layer of basal cells; syncytia with inclusion bodies sometimes present) and a score of 5 represents very severe changes (same as a score of 4, except that the mucosa has no residual epithelium, and syncytia with inclusion bodies are rarely found).

For the statistical analysis of the data, GraphPad Prism 9.0.0 (GraphPad Software, San Diego, CA, USA) was used. Kruskal–Wallis test and Dunn’s multiple comparison test were used to analyze bodyweight data, clinical scores, viral genome load, anti-ILTV antibody titer, CD4+ and CD8+ cell fractions. The percentage of remaining animals following the euthanasia of chickens that reach humane end points were analyzed using the log rank (Mantel–Cox) test and Gehan–Breslow–Wilcoxon test. The group differences were considered significant at *p* ≤ 0.05.

## 3. Results

### 3.1. HVT Genome Loads

**Figure 1:**
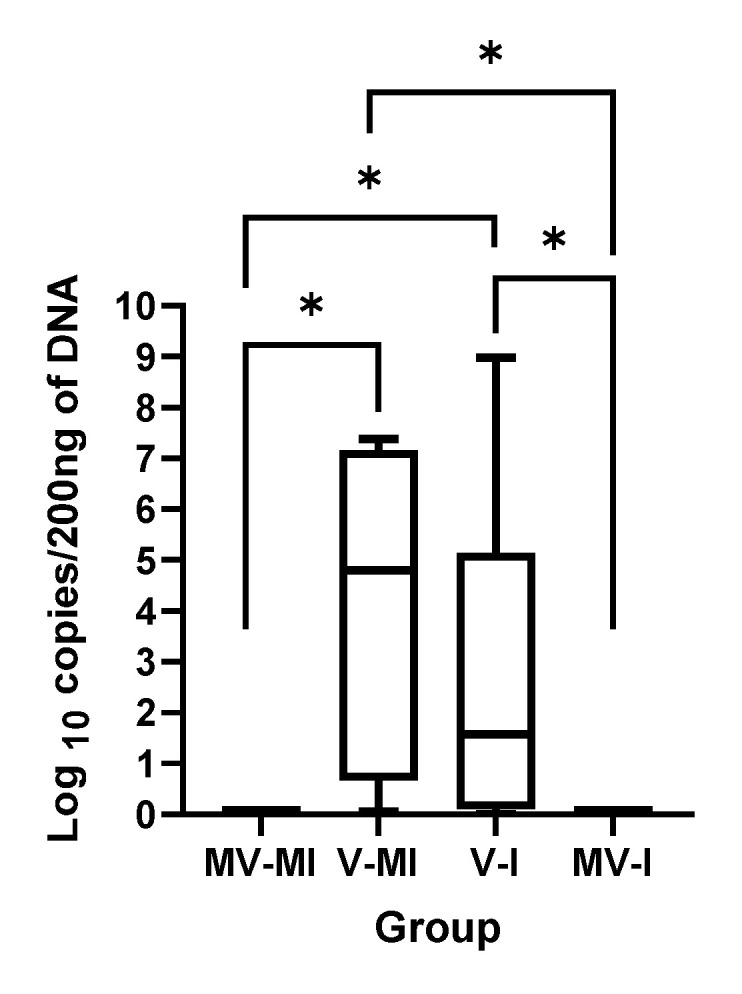
rHVT-LT genome load in spleen at 5 weeks post-vaccination (14 dpi) as an indicator of rHVT-LT vaccine successful application. The rHVT-LT genome loads targeting the gB gene of the HVT were quantified using the qPCR technique. The Kruskal–Wallis test followed by Dunn’s multiple comparison test was used to compare group differences. * = *p* < 0.05.

The HVT genome loads in spleen samples obtained at 14 dpi are illustrated in Figure 1 as an indication of correct rHVT-LT vaccine application. The HVT genome could be quantified in all spleen samples from the vaccinated groups (V-MI and V-I). No HVT genome was detectable in the spleens of the mock-vaccinated groups (MV-MI and MV-I). There was no statistically significant difference between the vaccinated groups (*p* > 0.05).

### 3.2. Percentages of Remaining Animals Following Euthanasia of Chickens Reaching Humane Endpoint

**Figure 2:**
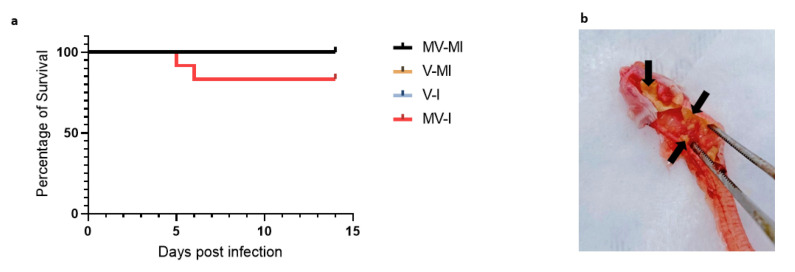
Percentage of survival (**a**) Percentages of remaining chicken following euthanasia of chickens reaching humane end point. Log rank (Mantel–Cox) test and Gehan–Breslow–Wilcoxon test were performed to identify group differences. (**b**) Post-mortem examination of trachea of a chicken euthanized at 6 dpi with fibrinous exudate in trachea (black arrows).

The clinical signs were scored, the chicken accumulating a critical clinical score were humanely euthanized and the remaining animals are illustrated as percentages of survival in Figure 2a. No difference was found between the groups’ percentage of survival. Two chickens were euthanized, one at 5 and the other at 6 dpi. In the post-mortem examination of the one chicken that reached endpoint at 6 dpi, we observed a fibrinous exudate buildup in the lumen of the trachea (Figure 2b).

### 3.3. Clinical Signs

**Figure 3:**
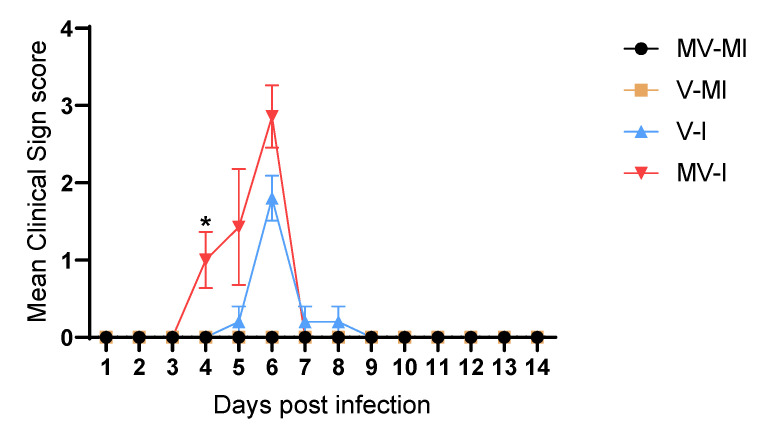
The observed mean clinical scores during experimental period. The mean clinical scores are given, and the error bars represent the standard error of means (SEM). Kruskal–Wallis test followed by Dunn’s multiple comparison test was performed to compare mean clinical scores of groups. * = *p* < 0.05.

None of the mock-infected animals (vaccinated and unvaccinated) had clinical signs throughout the experimental period. At 4 dpi, the mean clinical scores of the MV-I group were significantly higher than in the MV-MI (*p* < 0.05), V-MI (*p* < 0.05) and V-I (*p* < 0.05) groups. The peak of clinical signs was observed at 6 dpi in both ILTV-infected groups. At 6 dpi, the V-I groups had higher clinical scores than MV-MI (*p* < 0.05) and V-MI (*p* < 0.05) groups. The MV-I group also had higher clinical scores than the MV-MI (*p* < 0.05) and V-MI (*p* < 0.05) groups. However, at the peak of the disease (6 dpi) there was no difference between the infected groups (Figure 3, *p* > 0.05).

The clinical signs observed in the V-I group were open beak respiration, depression and conjunctivitis. On the other hand, the MV-I group exhibited ruffled feathers, depression, constant open beak respiration, severe dyspnea marked by gasping, bodyweight loss and conjunctivitis.

### 3.4. Bodyweight

**Figure 4:**
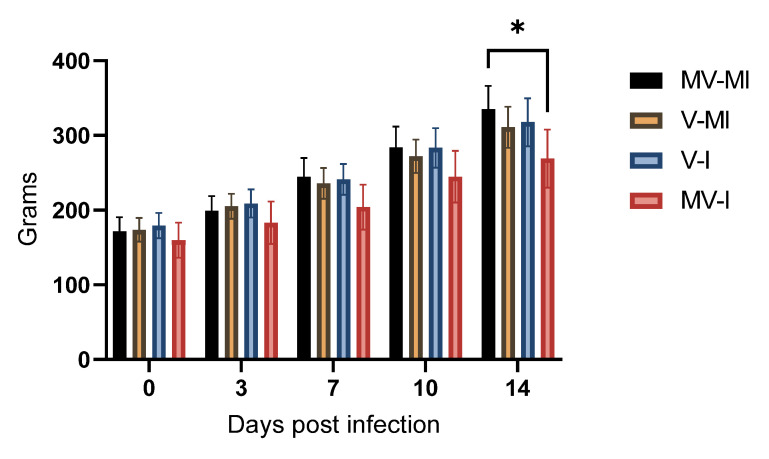
Bodyweight gains of experimental chickens at 0, 3, 7, 10 and 14 days post-infection (dpi). Data are presented as mean, and the error bars represent the standard error of means (SEM). Kruskal–Wallis test followed by Dunn’s multiple comparison test was used to identify group differences. * = *p* < 0.05.

Mean bodyweights at 0, 3, 7, 10 and 14 dpi are shown in Figure 4. At 14 dpi, the MV-I group had significantly lower mean bodyweights than the MV-MI group (*p* < 0.05). However, the difference between the V-I and MV-I groups was not significant (*p* > 0.05).

### 3.5. ILTV Genome Loads

#### 3.5.1. ILTV Genome Loads in Oropharyngeal Swabs

**Figure 5:**
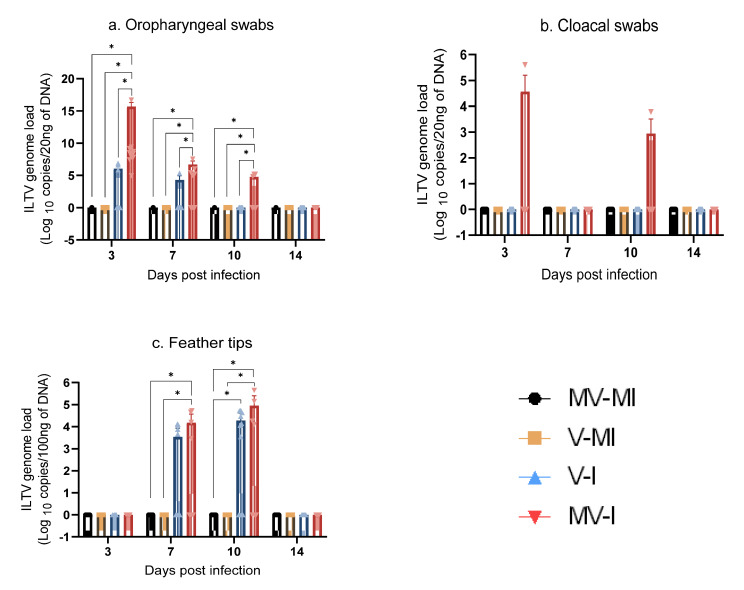
ILTV genome loads quantified targeting the PK gene at 3, 7, 10 and 14 days post-infection (dpi). The bars represent the mean, and error bars indicate the standard error of mean (SEM). Kruskal–Wallis test followed by Dunn’s multiple comparison test was used to identify group differences. (**a**) ILTV genome loads in oropharyngeal swabs; (**b**) ILTV genome loads in cloacal swabs; (**c**) ILTV genome loads in feather tips. * = *p* < 0.05.

The ILTV genome loads in oropharyngeal swabs are illustrated in Figure 5a. As expected, the mock-infected groups were negative for the ILTV genome at the observed time points. At 3, 7 and 10 dpi, the ILTV genome loads in the MV-I group were significantly higher than those observed in the V-I (*p* < 0.05), V-MI (*p* < 0.05) and MV-MI groups (*p* < 0.05). At 14 dpi, none of the ILTV-infected groups were positive for the ILTV genome.

#### 3.5.2. ILTV Genome Loads in Cloacal Swabs

The ILTV genome loads in cloacal swabs are illustrated in Figure 5b. Only one bird in the MV-I group was positive at 3 and 10 dpi. There were no statistically significant differences between the groups at any time point (*p* > 0.05).

#### 3.5.3. ILTV Genome Loads in Feather Tips

The ILTV genome loads in feather tips are illustrated in Figure 5c. None of the groups were positive for ILTV genome at 3 and 14 dpi. At 7 dpi, the MV-I group had significantly higher ILTV loads than the MV-MI (*p* < 0.05) and V-MI groups (*p* < 0.05). At 10 dpi, the V-I group had significantly higher ILTV loads than the MV-MI (*p* < 0.05) and V-MI groups (*p* < 0.05). The MV-I group also had a significantly higher ILTV loads at 10 dpi compared to the MV-MI (*p* < 0.05) and V-MI (*p* < 0.05) groups. There was no difference between the V-I and MV-I groups at 7 and 10 dpi (*p* > 0.05).

#### 3.5.4. ILTV Genome Loads in Trachea and Lungs

**Figure 6:**
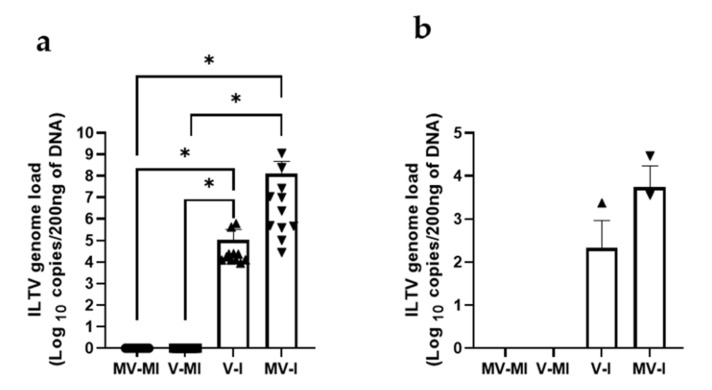
ILTV genome loads in trachea (**a**) and lungs (**b**) at 14 dpi. The quantification of ILTV genome loads was performed targeting the ILTV PK gene using qPCR assay. Mean genome load is plotted in log_10_ scale and represented in bars with standard error of means (SEM). Kruskal–Wallis test followed by Dunn’s multiple comparison test was performed to identify group differences. * = *p* < 0.05.

**Figure 7:**
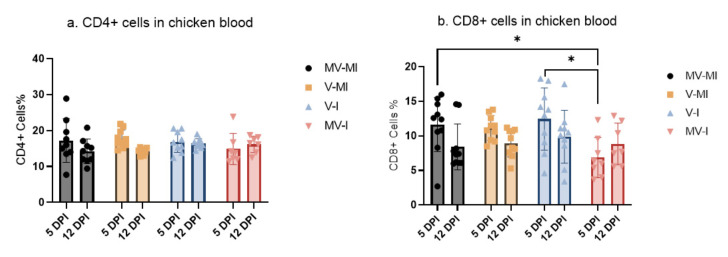
Percentage of (**a**) CD4+ and (**b**) CD8+ T cells in peripheral blood mononuclear cells (PBMCs) at 5 and 12 dpi. Mean percentage of CD4+ and CD8+ T cells is given, and error bars represent the standard error of means (SEM). Kruskal–Wallis test was performed followed by Dunn’s multiple comparison test to identify the group differences. * = *p* < 0.05.

The ILTV genome loads in trachea and lungs quantified at 14 dpi are illustrated in Figure 6a,b, respectively. The MV-I had higher ILTV genome loads in the trachea when compared to the MV-MI (*p* < 0.05) and V-MI (*p* < 0.05) groups. Similarly, the V-I group had significantly higher ILTV genome loads in the trachea when compared to the MV-MI (*p* < 0.05) and V-MI (*p* < 0.05) groups. However, ILTV genome loads in the trachea between the MV-I group and the V-I group showed no difference (*p* > 0.05). Although we observed a similar pattern of ILTV genome loads in the trachea and lungs (Figure 7a,b), there were no significant differences in the ILTV genome loads in the lungs between the groups (*p* > 0.05).

### 3.6. Peripheral Blood CD4+ and CD8+ T Cells

The results of the flow cytometry analysis of peripheral blood CD4+ and CD8+ T cells are illustrated in Figure 7a,b, respectively. The percentage of CD8+ T cells in the MV-I group at 5 dpi was significantly lower when compared with the MV-MI (*p* < 0.05) and V-I (*p* < 0.05) groups. There was no statistical difference in CD8+ T cell percentage at 12 dpi between the groups. Additionally, we did not observe group differences in CD4+ T-cell percentages in any of the observed time points (*p* > 0.05).

### 3.7. Anti-ILTV Antibody Response

**Figure 8:**
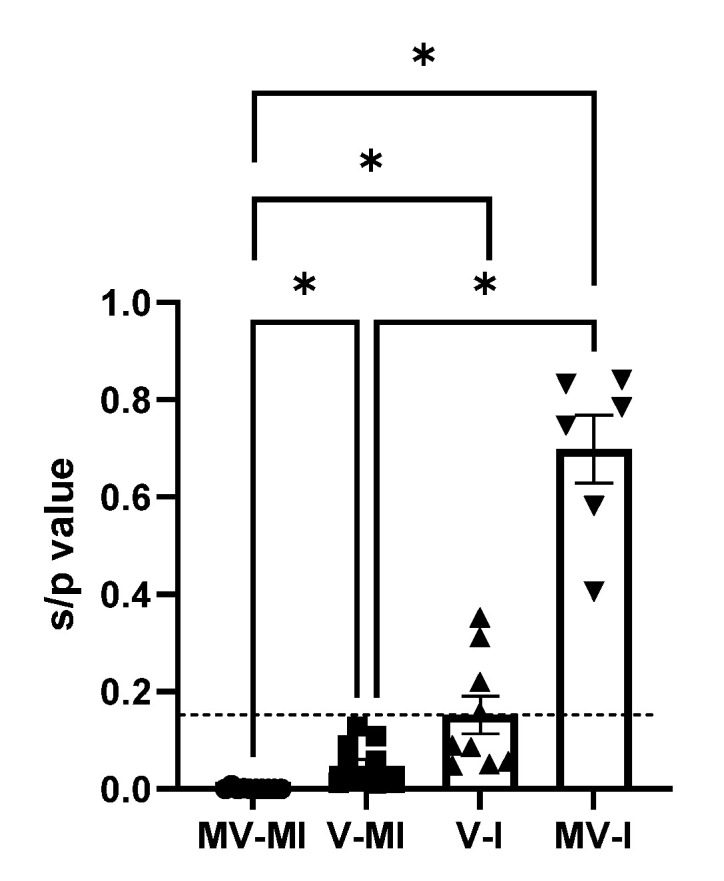
Serum sample to positive value (*s*/*p*) at 14 dpi. The bars represent mean; the geometric figures, the individual values; and the error bars, the standard error of mean (SEM). The horizontal discontinuous line represents the cut-off value of negative to positive serological diagnostic results, corresponding to 0.15. Kruskal–Wallis test followed by Dunn’s multiple comparison test was used to compare the group differences. * = *p* < 0.05.

The *s*/*p* values obtained by ELISA at 14 dpi are illustrated in Figure 8. The MV-I group had a significantly higher *s*/*p* value than the MV-MI group (*p* < 0.05) and the V-MI group (*p* < 0.05). The V-I group had a statistically higher *s*/*p* value than the MV-MI (*p* < 0.05). Neither the V-MI nor the MV-MI had values above the cut-off point (0.15). There was no statistically significant difference between the two infected groups.

### 3.8. Histology

**Figure 9:**
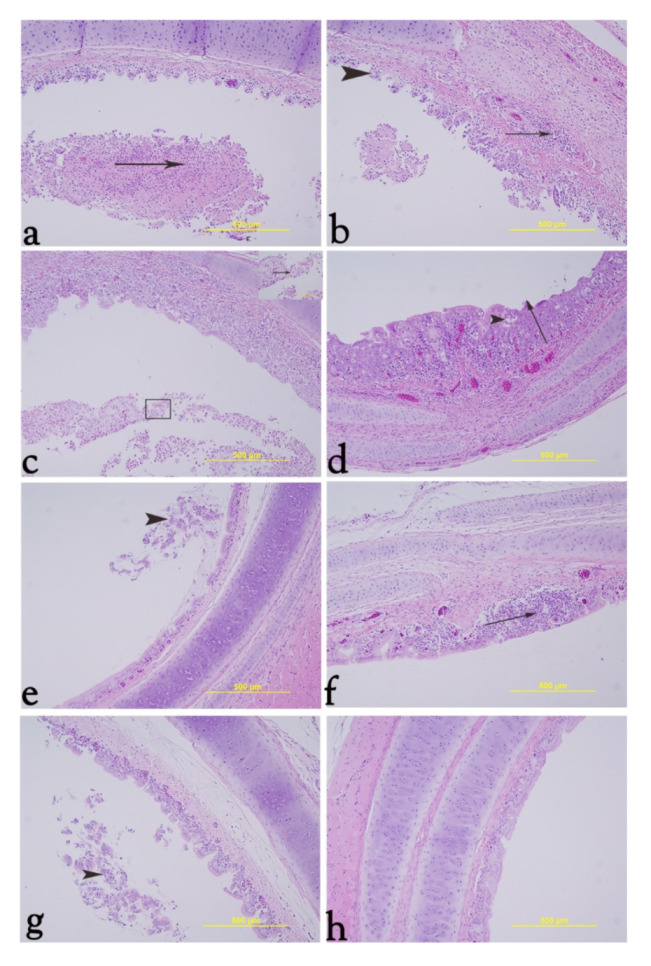
Representative histology images of trachea at 14 dpi and quantitative tracheal histological score. (**a**): Black arrow represents fibrinohemorrhagic membrane containing cellular exudates. (**b**): Black arrow represents mononuclear cell infiltration and hyperemia; head arrow indicates projection of the underlying connective tissue into the lumen. (**c**): Eosinophilic intranuclear viral inclusion inside sloughed epithelial cells around heterophilic infiltrates (inset). (**d**): Vacuolar spaces containing cellular debris and mononuclear cell infiltration with hyperemia in l. propria (head arrow); the epithelium lining exhibited squamous metaplasia (black arrow). (**e**): Black head arrow represents cuffs of sloughed epithelial cells in the tracheal lumen. (**f**): Black arrow represents mononuclear cell infiltration, hyperemia and edema. (**g**): Black head arrow represents intraluminal sloughed epithelial cells. (**h**): Normal trachea without pathological lesion.

In general, the most severe microscopic lesions were detected in the trachea after the exposure of chickens to ILTV isolate in comparison to the V-I group and the controls (Table 2). It was clear that there were variations in tracheal lesion scores among birds of the same group (Figure 9). The MV-MI group had very minimal pathological lesions (Figure 9) with a mean tracheal lesion score of 0.

Initially, the MV-I group was associated with wide patches of necrosis, desquamation of the epithelial lining with deciliation, intraluminal fibrinohemorrhagic and cellular exudates (heterophils and necrotic epithelial cells); the lamina propria was infiltrated with mononuclear cells; and the underlying connective tissue was projected into the tracheal lumen (tracheal lesion score = 5) (Figure 9a,b). In some cases, there were infiltrated heterophils together with mononuclear cells in the lamina propria, and the tracheal lumen was filled with abundant necrotic epithelial cells; fibrin, heterophils and intranuclear viral inclusion could be observed in some epithelial cells (tracheal lesion score = 4) (Figure 9c). Additionally, the epithelial lining revealed squamous metaplasia and vacuolar degeneration with the formation of vacuolar spaces containing cellular debris, the sub epithelial tissue was infiltrated with mononuclear cell infiltration (tracheal lesion score = 3) (Figure 9d).

In the V-I group, the trachea showed necrosis with desquamation of the epithelial lining and ciliary loss and intraluminal ciliated cuffs of necrotic cells admixed with mucous, and hyperemia was observed in the lamina propria (tracheal lesion score = 3) (Figure 9e). Focal mononuclear cell infiltrations, hyperemia and edema were also observed (tracheal lesion score = 2) (Figure 9f).

The V-MI group showed necrosis and sloughed epithelium with ciliary loss in some areas and cuffs of desquamated ciliated epithelial cells in the tracheal lumen (tracheal lesion score = 3) (Figure 9g).

**Figure 10:**
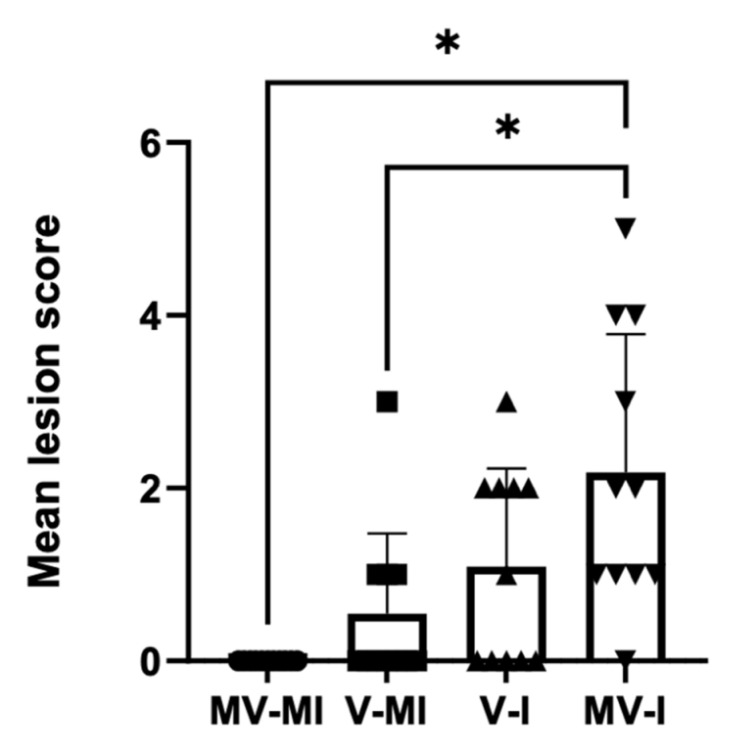
The mean lesion scores are given, and the error bars represent the standard error of means (SEM). Kruskal–Wallis test followed by Dunn’s multiple comparison test was performed to compare mean clinical scores of groups. * = *p* < 0.05.

The quantitative tracheal histological scores are given in Figure 10.

## 4. Discussion

Several of the expected outcomes of vaccination in poultry are as follows: protection against clinical disease, a reduction in susceptibility to infection and reduction in viral shedding [46]. The vaccine protection of a rHVT-LT-based vaccine was assessed after challenge with a field strain of ILTV AB-S63 by comparing weight gain, histopathological changes, and clinical sign scores. The rHVT-LT vaccine induced a level of protection in our study. This was demonstrated by a slower onset of clinical signs in V-I group. At the peak of the clinical signs (6 dpi), there was no difference between the infected groups. Previous studies have shown that the recombinant vaccine administered to chicken that were challenged between 28 days to 35 weeks of age appears to reduce clinical signs [32,35,36,37,38]. Nonetheless, protection elicited by the rHVT-LT vaccine administered in chickens challenged at 74 weeks of age was minimal [38]. In the case of chickens given rHVT-LT that contained gB only and challenged at a younger age (21 days of age), the protection against clinical disease was partial, with 67% of vaccinated birds protected [32].

We evaluated ILTV genome loads as an indicator of ILTV shedding in feather tips, oropharyngeal and cloacal swabs. In agreement with our observation of decreased viral shedding via the oropharyngeal route, previous studies have demonstrated that the rHVT-LT vaccine can significantly reduce viral shedding (3 dpi) as indicated by viral loads determined using tracheal swabs [35,36,37]. However, it is worth noting that some of these studies did not record significant reductions in viral loads in tracheal swabs at 7 dpi [35,36] or beyond this time point [36], contradicting our findings. Although it is difficult to explain the difference in respiratory tract viral shedding between the current study and previous studies, it is possible that it may be related to the difference in time between vaccination and ILTV infection (21 days vs. 55–57 days). In support of this hypothesis, it has been shown that the vaccine induced immune response wanes by 57 days after vaccination [35]. Another study showed no significant viral reduction using tracheal swabs at 5 and 8 dpi in the rHVT-LT vaccinated group [34]. This study attributed the failure in protection efficacy due to incorrect in ovo delivery of the vaccine. It is also important to note that some of these studies used live attenuated vaccine-related ILTV strains as the challenge strain [32,34,35,38]. In our study, we used a Canadian origin genotype VI wild-type ILTV. One of the chickens of the MV-I group tested positive at 3 and 10 dpi. Previously, it has been shown that the ILTV is quantifiable in feather tips [39,47,48]. However, we did not observe that the rHVT-LT vaccine given at 1 day of age is effective in reducing ILTV genome loads in feather tips.

However, we observed that ILTV genome loads in oropharyngeal swabs were reduced in the vaccinated group; we did not observe a difference in ILTV genome loads in the lungs and trachea (14 dpi) between these two groups. Our observation agrees with the previous literature, which indicates that ILTV can persist at 14 dpi due to the reactivation of latent ILTV infection or viral residue from a previous lytic infection [49,50]. A similar trend was observed in the histopathological lesion scores with no difference in lesion scores between V-I and MV-I groups. The V-MI group presented some mild lesions on trachea. However, it seems highly unlikely that this was caused by the vaccine, as the birds were vaccinated at 1 day of age and the tissues were collected 5 weeks later.

It is known that cellular immune response plays an essential role in vaccine-induced antiviral immunity and viral infections [51,52]. In the case of ILTV infections, it is well documented that cell-mediated immunity is the primary immune response [8,53,54]. While there are no prior studies that evaluate the dynamics of T cells in PBMCs after ILTV infection or rHVT-LT vaccination in chickens, there are studies that have investigated T-cell response in the context of other respiratory viruses, such as Newcastle disease virus (NDV) and avian influenza virus (AIV) [43,55,56,57]. Our experimental findings suggested that the rHVT-LT vaccine promotes the presence of CD8+ T in early infection (5 dpi). This could explain the lower clinical sign score in the V-I at 4 dpi. Similarly, one study showed a significant increase in CD8+ T cells at 2, 3 and 7 dpi when compared to the non-vaccinated and NDV-infected group [56]. In a different context, a significant increase in CD8+ T cells in vaccinated chickens has also been shown [43]. On the other hand, researchers observing the cellular and humoral immune response after challenge with a low pathogenic AIV found a significant increase in CD4+ T cells in the challenged group at 14 dpi [55]. However, in our study, we did not observe a difference in CD4+ T at any of the sampling time points among the groups.

In the current study, we observed that the *s*/*p* value of MV-I chickens did not exhibit a statistically significant difference when compared to V-I chickens. Additionally, we observed that none of the birds in the vaccinated and mock-infected group had *s*/*p* values that reached the cut-off value. The role of antibody-mediated immune response against ILTV infection is unclear [8,54,58,59]. It is also important to note that previous studies have demonstrated that the gG of ILTV can inhibit T-cell responses and favor the humoral immune response as an immune evasion strategy [60,61].

## 5. Conclusions

In conclusion, the rHVT-LT vaccine diminishes viral shedding and augments the peripheral blood CD8+ T-cell response. Nonetheless, it does not reduce the clinical signs at the peak of the disease or prevent ILTV genome loads or lesions in respiratory tissues. Additional studies with contact birds are needed to assess if a reduction in viral shedding might reduce the transmission of ILTV.

## Figures and Tables

**Table 1:** Experimental design.

Group	n	Vaccination (1 Day of Age)	ILTV Infection (3 Weeks of Age)
MV-MI ^1^	11	0.2 mL of vaccine diluent	0.2 mL of PBS
V-MI ^2^	11	0.2 mL of Innovax^®^ ILT vaccine	0.2 mL of PBS
V-I ^3^	11	0.2 mL of Innovax^®^ ILT vaccine	0.2 mL of AB-S63 ILTV
MV-I ⁴	11	0.2 mL of vaccine diluent	0.2 mL of AB-S63 ILTV

^1^ Mock vaccinated and mock infected; ^2^ vaccinated and mock infected; ^3^ vaccinated and infected; ⁴ mock-vaccinated and infected.

**Table 2:** The mean microscopic lesions detected in 4 groups.

Microscopic Lesions	MV-I ^1^	V-I ^2^	V-MI ^3^	MV-MI ^4^
Lamina epithelialis and intratracheal lumen:				
Necrotic desquamated epithelial cells and deciliation	8/11	3/11	1/11	0/11
Cellular exudate (heterophils, necrotic epithelium, erythrocytes)	3/11	0/11	0/11	0/11
Fibrinohemorrhagic exudate	2/11	0/11	0/11	0/11
Eosinophilic intra nuclear viral inclusions	2/11	0/11	0/11	0/11
Lamina propria:				
Mononuclear cell infiltration	8/11	2/11	3/11	0/11
Hyperemia	3/11	1/11	2/11	1/11

^1^ Mock vaccinated and infected; ^2^ vaccinated and infected; ^3^ vaccinated and mock infected; ⁴ mock vaccinated and mock infected.

## Data Availability

Not applicable.
